# Mosaic Turner Syndrome Presenting with a 46,XY Karyotype

**DOI:** 10.1155/2019/3719178

**Published:** 2019-04-11

**Authors:** Melody Rasouli, Katherine McDaniel, Michael Awadalla, Karine Chung

**Affiliations:** ^1^University of Southern California Keck School of Medicine, Los Angeles, CA, USA; ^2^Department of Obstetrics and Gynecology, University of Southern California Keck School of Medicine, Los Angeles, CA, USA

## Abstract

Although Turner syndrome is most commonly associated with a 45,X genotype, other mosaic genotypes are present in approximately half of all cases. We describe a case of Turner syndrome with a 46,XY genotype by conventional 5-cell karyotype who was subsequently found to have a mosaic genotype of 18% 45,X and 82% 46,XY by 50-cell FISH analysis. Individuals with a mosaic 45,X/46,XY genotype have a variety of phenotypic presentations ranging from male to female which are not correlated with the percentage of mosaicism. Our case represents an extreme example where the genotype is predominately 46,XY and the phenotype typical of Turner syndrome.

## 1. Introduction

Turner syndrome is diagnosed in females based on clinical presentation combined with a genotype consisting of one normal X chromosome and complete or partial absence of the other X chromosome [1]. Patients with 45,X/46,XY mosaicism present with a variety of phenotypes ranging from most commonly mixed gonadal dysgenesis to others such as phenotypic males, genital ambiguity, Turner syndrome, and women with normal female secondary sex characteristics [2, 3]. Turner syndrome presents with bilateral streak gonads, whereas mixed gonadal dysgenesis describes those presenting with an absent or abdominal streak gonad on one side and a normal or dysgenic testis on the other. The phenotype in a 45,X/46,XY mosaic patient likely depends on the distribution of mosaicism percentage in different tissues which has been shown to differ between blood and gonadal tissue [4]. This case is an example where the dominant mosaic genotype in the blood (46,XY) is discordant with the Turner syndrome phenotype.

## 2. Case Presentation

A 38-year-old gravida 1 para 0010 Russian female presented with irregular menses every 2-3 months and a 15-year history of infertility. Prior to presenting to our institution, she was seen by a fertility specialist in Russia where a karyotype analysis was performed. A copy of the result was not available for review by our clinicians, but the patient believed that she was found to have a 46,XY karyotype. The patient was unaware of any other relevant lab results. The patient underwent menarche at the age of 15 and had irregular menses every 2-3 months since then. She had an early first trimester spontaneous abortion which was detected with a positive home urine pregnancy test without clinical ultrasound or pathological confirmation. She had a history of a laparoscopic appendectomy with a concurrent right salpingectomy. She did not have any other significant medical or family history. Specifically she had no family history of irregular menses, infertility, or premature ovarian failure.

On exam, she was 160 cm tall and weighed 55 kg with a BMI of 23. Her vital signs were normal and she had normal female secondary sex characteristics with Tanner stage V breast development, Tanner stage V pubic hair growth, a normal vagina and cervix, and no hirsutism or clitoromegaly. She was without short stature, scoliosis, high palate, hearing loss, short or webbed neck, shield chest, cubitus valgus, shortened fourth metacarpals or metatarsals, genu valgum or varum, or Madelung deformity of the forearm and wrist.

Laboratory studies showed premature ovarian insufficiency with a follicle stimulating hormone level of 104.9 mIU/mL, a luteinizing hormone level of 35.5 mIU/mL, an estradiol level of < 5 pg/mL, and a total testosterone level of <12 ng/dL. Liver function and thyroid function tests were within normal limits. A peripheral blood karyotype analysis of 5 cells at a 400-550 band resolution showed a normal 46,XY male karyotype (Chromosome Analysis Blood, Quest Diagnostics). Although this karyotype is consistent with complete gonadal dysgenesis (Swyer syndrome), the patient's clinical history of breast development and menses did not fit this diagnosis. A FISH analysis was performed on 50 cells for evaluation of SRY and the X centromere to evaluate for possible Swyer syndrome or low-level mosaicism. This showed 41 cells with 46,XY and 9 cells with 45,X (FISH SRY/X Centromere, Quest Diagnostics) which was clinically correlated to a diagnosis of mosaic Turner syndrome.

Sonographic examination revealed a small uterus measuring 4.4 × 2.3 × 1.2 cm, a right ovary measuring 1.4 × 1.2 × 0.9 cm with two simple cysts measuring 8 mm and 9 mm, a left ovary measuring 1.3 × 0.9 × 0.8 cm, and a 6 mm endometrial echo complex. A CT scan of the abdomen and pelvis showed normal kidneys. An echocardiogram was performed and showed no cardiac anatomical abnormalities. A dual-energy X-ray absorptiometry (DEXA) scan showed lumbar osteoporosis with a T-score of -3.5.

**Figure 1:**
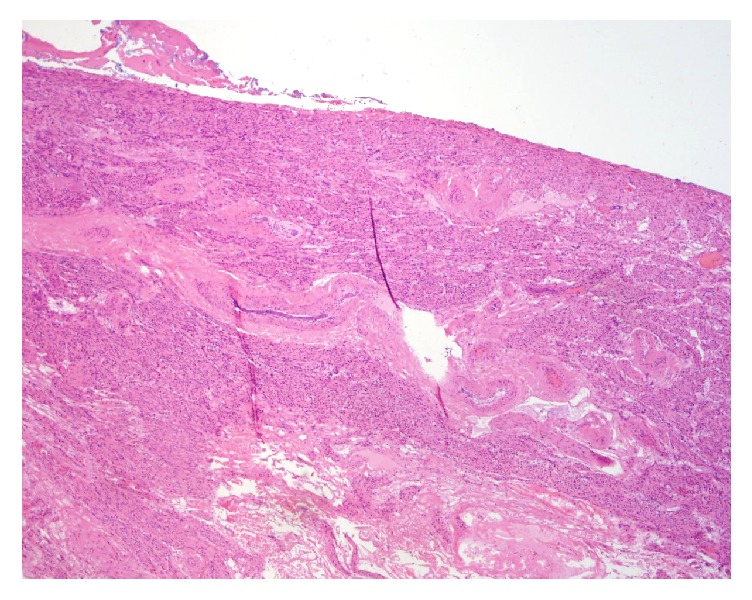
Pathology of the right and left ovaries shows hypoplastic ovarian tissue with fibrotic stroma and an absence of follicles.

**Figure 2:**
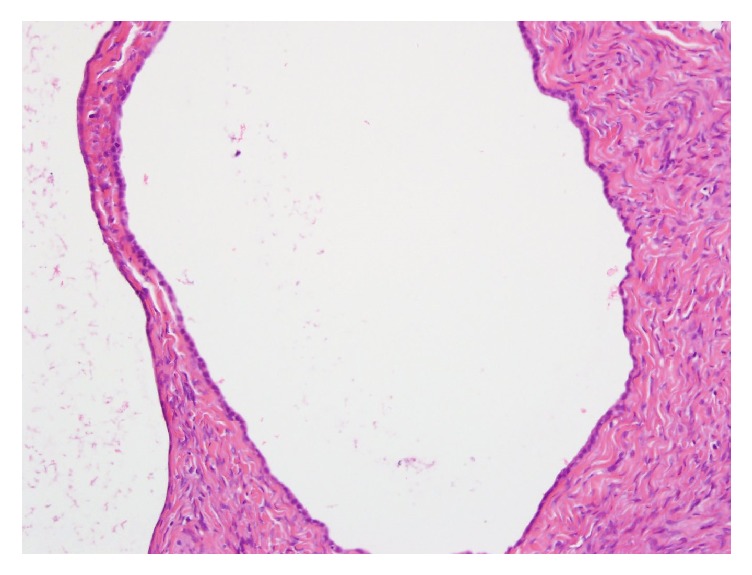
Pathology of the right ovary shows benign serous cysts.

Due to the increased risk of gonadoblastoma, the patient was offered and accepted laparoscopic bilateral gonadectomy and left salpingectomy (her right fallopian tube was surgically absent) with pelvic washings. On pathologic review, the bilateral gonads were found to possess hypoplastic ovarian tissue (Figure 1) with two small right ovarian serous cysts (Figure 2) and no evidence of malignancy. For her osteoporosis, she was prescribed calcium and vitamin D supplementation and she preferred to be on cyclic combined oral contraceptives rather than standard hormone replacement therapy. She was counseled that pregnancy is an option for her through in vitro fertilization with donor eggs and she intends to pursue this when ready for family building. She was counseled that bisphosphonates are not recommended in women considering future pregnancy and referred to medical endocrinology for treatment of osteoporosis with other non-bisphosphonate medications.

## 3. Discussion

Turner syndrome is associated with multiple chromosome abnormalities including 45,X and 45,X/46,XX and 45,X/47,XXX and 45,X/46,XY. The 45,X/46,XY genotype accounts for approximately 10-12% of cases of Turner syndrome [1]. In a report of 76 prenatally diagnosed cases of 45,X/46,XY mosaicism, 75 had male appearing external genitalia and only one had female genitalia [5]. In a series of 27 postnatally diagnosed cases of 45,X/46,XY mosaicism, 18 were male (11 with mixed gonadal dysgenesis) and 9 had Turner syndrome [2]. Turner syndrome with 45,X/46,XY low-level mosaicism may not be detected on standard karyotype and FISH analysis of larger numbers of cells can be useful for diagnosis.

The American College of Medical Genetics (ACMG) provides guidelines for karyotyping procedure specific to Turner syndrome. The College recommends karyotyping a minimum of 30 cells due to the high incidence of mosaicism, unless mosaicism is encountered within the first 20 cells. When there is a high clinical suspicion of Turner syndrome in a patient with a 46,XX karyotype, cytogenetic study of a second tissue type (such as skin biopsy for cell culture or buccal smear for FISH) is advised. Additionally, given the risk of gonadoblastoma with occult Y chromosome mosaicism, the ACMG recommends 200 cell FISH analysis to probe for the X and Y centromeres when 30-cell karyotype results in a nonmosaic 45,X karyotype [6].

Women with Turner syndrome who possess Y chromosome material have an increased risk of germ cell tumors such as gonadoblastoma and dysgerminoma. A national cohort study that included 211 of these patients estimated that, by age 25, the cumulative risk of gonadoblastoma is 7.9% (95% CI 3.1-19.0) [7]. Although rates of gonadoblastoma in Turner syndrome patients with Y chromosome material vary by study from as low as 4% to as high as 60%, current evidence suggests that the rate is approximately 10%. Prophylactic gonadectomy is recommended at the time of diagnosis for patients with Turner syndrome and Y chromosome material such as 45,X/46,XY mosaicism [1].

Turner syndrome patients with any genotype should undergo standard testing and treatment for cardiovascular, renal, metabolic, endocrine, vision, hearing, and bone mineral density abnormalities [1]. If premature ovarian failure is diagnosed, hormone replacement therapy is indicated until the typical age of menopause to induce puberty and secondary sexual characteristics, stimulate uterine growth, and prevent bone loss. It is generally recommended to begin treatment with low-dose E_2_ (3-7*μ*g/day of transdermal E_2_ or 0.25mg oral E_2_ per day) at age 11 or 12 and incrementally increase the dose to adult doses (50-150*μ*g/day of transdermal E_2_ or 2-4mg oral E_2_ per day) over 2 to 3 years. Transdermal estradiol is preferred, followed by oral or intramuscular estradiol. Oral ethinyl estradiol is not recommended, unless other options are unavailable or for issues relating to patient preference or compliance as was the case for the patient presented here. A progestin is added once breakthrough bleeding occurs or 2 years after E_2_ is begun to decrease the risk of endometrial hyperplasia. Progestin can be administered orally in a cyclic pattern with E_2_, orally continuously with E_2_, or in the form of a progestin containing intrauterine device [8].

Future fertility is an important consideration for patients with Turner syndrome. Accurate and early diagnosis of 45,X/46,XY mosaicism can allow for counseling about reproductive potential and pursuing pregnancy with in vitro fertilization with donor egg and/or gestational surrogacy. Successful pregnancy outcomes have occurred in patients with 45,X/46,XY mosaicism as well as 46,XY gonadal dysgenesis following oocyte donation and in vitro fertilization, although most of the reported cases were delivered by cesarean section [9–13]. Although this patient's uterus measured only 4.4 × 2.3 × 1.2 cm on ultrasound, there is no contraindication to pregnancy due to uterine size. Uterine size is likely a result of low estrogen status rather than an indication that the uterus is unfit to carry a pregnancy to term.

In summary, this case demonstrates that Turner syndrome with low level mosaicism may be missed by conventional karyotype. Some females diagnosed with Swyer syndrome may actually have Turner syndrome with low level mosaicism. Approximately 70-80% of patients diagnosed with Swyer syndrome do not have SRY mutations [14, 15], and Turner syndrome with low level mosaicism may be the actual cause of gonadal dysgenesis in some of these patients. In cases where conventional karyotype results do not closely match the clinical presentation, FISH analysis for low level mosaicism may be informative.
